# New WHO odontogenic tumor classification: impact on prevalence in a population

**DOI:** 10.1590/1678-7757-2019-0067

**Published:** 2019-11-11

**Authors:** Bianca Caroline Figueiredo Bianco, Felipe Fornias Sperandio, João Adolfo Costa Hanemann, Alessandro Antônio Costa Pereira

**Affiliations:** 1 Universidade Federal de Alfenas, Instituto de Ciências Biomédicas, Departamento de Patologia e Parasitologia. Alfenas, MG, Brasil.; 2 Universidade Federal de Alfenas, Faculdade de Odontologia, Departamento de Clínica e Cirurgia, Alfenas, MG, Brazil.

**Keywords:** Oral pathology, Odontogenic tumors, Biopsy, Neoplasia

## Abstract

**Objectives:**

This study approaches the history of reclassifications and redefinitions around the odontogenic keratocyst (OK), as proposed by the World Health Organization (WHO), and aims to understand the impact of those changes on the prevalence and epidemiology of odontogenic tumors (OTs).

**Methodology:**

Cases of OTs diagnosed in an Oral Pathology service between January 1996 and December 2016 were reviewed. Demographic data of patients such as age, gender and site of lesions were retrieved from their respective records.

**Results:**

Within the studied period, 7,805 microscopic reports were elaborated and 200 (2.56%) of these were diagnosed as OTs. Out of these 200, between 1996 and 2005, prior to the 2005 WHO classification, there were 41 (20.5%) OTs cases, being odontoma the most frequent (23; 56.09%), followed by ameloblastoma (8; 19.51%) and myxoma (03; 7.31%). Between 2006 and 2016, after the previous 2005 WHO classification there were 159 (79.5%) OTs, being odontogenic keratocystic tumor (KCOT) the most frequent (68; 42.76%), followed by odontoma (39; 24.52%) and ameloblastoma (21; 13.20%).

**Conclusions:**

As of today, the most recent WHO classification to be followed brings KCOT back to the cyst category, which will impact on the prevalence and epidemiology of OTs; thus, this study was able to identify a considerable increase (287.80%) in the prevalence of OTs when the 2005 WHO classification was utilized. Despite being an important academic exercise, classifying odontogenic lesions and determining whether to place the odontogenic keratocyst in a cyst or tumor category is crucial to establish the correct diagnosis and treatment to follow, whether by oral medicine or oral surgery specialist, or by the general practitioner.

## Introduction

The first consensus about odontogenic tumors (OTs) classification resulted from a five-year study assembled by the World Health Organization (WHO) and was published in 1971, being accepted as the first international categorization for those tumors. Since then, OTs grouping has been an academic exercise for the Oral Medicine/Oral Pathology specialty.^1^ The classifications published by the WHO reflect the current status of comprehension of OTs; adaptations to that classification as well as updates become necessary as clinical and scientific experiences accumulate. Although such publications are based on specialists' opinions, they may be potentially contested by some pathologists. Nevertheless, it is recommended that all professionals employ and follow the presented categorization aiming at international standardization, given that all oral pathologists could benefit from it.^2^

The 1971 classification brings OTs as “Neoplasms and tumors related to odontogenic tissues”. Such edition classifies the odontogenic keratocyst under “Epithelial Cysts”, specifically odontogenic cysts under development and termed as primordial cyst or simply keratocyst. Nonetheless, odontogenic keratocyst presents a single form of developed odontogenic cyst and deserves special attention due to its pathological characteristics and specific clinical behavior.^2^^,^^3^ The 1992 classification continued to include OTs as “Neoplasms and other tumors related to odontogenic tissues” and the odontogenic keratocyst still as odontogenic cysts under development, but its name was changed to odontogenic keratocyst (OK).^4^ The 2005 WHO edition defined OTs as a group of heterogenous lesions that could vary from hamartomatous or neoplastic proliferations to benign neoplasms or malignant tumors with metastatic potential.^5^ Such edition omitted the classification of odontogenic cysts and reclassified and redefined OK to keratocystic odontogenic tumor (KOT).

The last published edition by the WHO outlines OTs as rare tumors – since these constitute only 1% of all oral tumors –, as well as benign entities that somehow may present an aggressive behavior and high recurrence rates.^6^ The 2017 edition places odontogenic cysts back to OTs and now classifies KOT as a cyst, also terming it odontogenic keratocyst.^6^ Considering that this is a common lesion, it is evident that reclassification and redefinition by this entity – both for tumor and cyst – causes a significant increase in the frequency and prevalence of OTs, as well as the ranking order among OTs. Other lesions that were included or excluded from 2017's classification could also influence the OTs epidemiology, less significantly than OK, as they are notably rarer. For the 2017 classification, the sclerosing odontogenic carcinoma, odontogenic carcinosarcoma, primordial odontogenic tumor and cemento-ossifying fibroma were included. The cystic calcifying odontogenic tumor was, relocated to the odontogenic cysts classification, whereas odonto ameloblastoma and ameloblastic fibro-odontoma were not considered to be single entities.

Thus, this study sought to approach the history of reclassifications and redefinitions around the odontogenic keratocyst (OK), as proposed by the WHO, as well as to understand the impact of those changes on the prevalence and epidemiology of odontogenic tumors (OTs) by assessing the collection of cases diagnosed in an Oral Pathology Service between January 1996 and December 2016.

## Methodology

### Sample selection

After approval by the Research Ethics Committee under the protocol 077338/2017, every single report of OTs that was diagnosed between January 1996 and December 2016 in the Oral Pathology laboratory of the Federal University of Alfenas (UNIFAL-MG) was assessed. Inclusion criteria comprised a microscopic final diagnosis of OT, including those in syndrome cases patients. There were no exclusion criteria.

### Demographic data assessment

The following demographic data of the patients were retrieved and analyzed: age, gender, skin color and OTs data: clinical aspect, symptomatology, radiographic, macroscopic size and clinical diagnostic hypotheses.

### Statistical Analysis

The prevalence of OTs was established considering the total number of biopsies performed in the laboratory from 1996 to 2016. Frequency and prevalence of OTs from January 1996 to December 2005 was compared to the frequency and distribution from January 2006 to December 2016. Statistical comparisons were made applying the Chi-square test (p<0.05 considered to be statistically significant).

## Results

Out of 7,805 cases diagnosed between the reported period, 200 (2.56%) were defined as OTs, being the prevalence before and after 2005 depicted in Table 1. Out of 200 cases between 1996 and 2005, 41 cases of OTs (20.5%) were found. Odontoma was the most frequent with 23 (56.09%) cases, followed by ameloblastoma with 8 (19.51%) cases, and myxoma with 03 (7.31%) cases.

**Table 1:** Distribution of OTs diagnosed before and after 2005 in a Brazilian Oral Pathology Center

Period	1996-2005	2006-2016
ODONTOGENIC TUMOURS		
KCOT	- (0,00%)	68 (42,76%)
Odontoma	23 (56,09%)	39 (24,52%)
Ameloblastoma	08 (19.51%)	21 (13,20%)
Myxoma	03 (7,31%)	10 (6,28%)
CCOT	- (0,00%)	11 (6,91%)
Odontogenic fibroma	01 (2,43%)	07 (4,40%)
Squamous Odontogenic Tumour	02 (4,87%)	- (0,00%)
CEOT	01 (2,43%)	01 (0,62%)
Cementoblastoma	02 (4,87%)	- (0,00%)
AOT	- (0,00%)	02 (1,25%)
Odontoameloblastoma	01 (2,43%)	- (0,00%)
Total	41 (100%)	159 (100%)

Between 2006 and 2016, after WHO's 2005 reclassification, we found 159 (79.5%) cases of OTs. During this period the most frequent lesion was KOT (68; 42.76%) cases, followed by odontoma (39; 24.52%) cases and ameloblastoma (21; 13.20%) cases. Of the 200 selected OTs cases, 108 (54%) affected males and 92 (46%) affected females, for a 1.17:1 male:female ratio. Considering that there was no statistically significant difference between groups, OTs affected both genders equally when this sample was analyzed. Distribution of OTs between genders is shown in Table 2.

**Table 2:** Distribution of OTs according to histology and gender in a Brazilian Oral Pathology Center

Gender	Male	Female	Total
ODONTOGENIC TUMOURS			
KCOT	37	31	68
Odontoma	35	27	62
Ameloblastoma	16	13	29
Myxoma	6	7	13
CCOT	5	6	11
Odontogenic fibroma	3	5	8
Squamous Odontogenic Tumour	-	2	2
CEOT	1	1	2
Cementoblastoma	2	-	2
AOT	2	-	2
Odontoameloblastoma	1	-	1
Total	108	92	200

Most cases related to the mandible, being 116 (58%) in this site (p<0.0001), which was followed by the maxilla with 68 (34%) cases, and 16 (8%) of those cases did not present this information. Distribution of OTs according to anatomical localization is shown in Table 3. Most cases are related to patients between 11 and 20 years of age, being 76 (38%) cases in patients at their second decade of life (p<0.0001), followed by the third decade, 25 (12.5%), and fourth decade, 18 (9%). Age was not disclosed in 31 (15.5%) cases. The distribution of OTs according to age is presented in Table 4.

**Table 3:** Distribution of OTs according to anatomical site in a Brazilian Oral Pathology Center

Site	Mandible	Maxilla	N.A.
ODONTOGENIC TUMOURS			
KCOT	49	18	1
Odontoma	29	28	5
Ameloblastoma	25	-	4
Myxoma	4	7	2
CCOT	1	10	-
Odontogenic fibroma	4	2	2
Squamous Odontogenic Tumour	1	1	-
CEOT	2	-	-
Cementoblastoma	1	1	-
AOT	-	1	1
Odontoameloblastoma	-	-	1
Total	116	68	16

**Table 4:** Distribution of OTs according to age in a Brazilian Oral Pathology Center

Age Range	n°	Percentage
0.0 |— 10.0	10	5,92%
11.0 |— 20.0	76	44,97%
21.0 |— 30.0	25	14,79%
31.0 |— 40.0	18	10,65%
41.0 |— 50.0	11	6,51%
51.0 |— 60.0	16	9,47%
61.0 |— 70.0	8	4,73%
71.0 |— 80.0	2	1,18%
81.0 |— 90.0	3	1,78%
91.0 |— 100.0	0	0,00%
TOTAL	169	100,00%

Prevalence of OTs before WHO's 2005 classification was 20.5%, whereas after the change in classification it grew to 79.5%, for a 287.80% increase (p<0.0001). If KOT had not been included after 2005, the prevalence of OTs from 2006 to 2016 would be of 45.5%, which would have been a 121.95% (p<0.05) increase.

## Discussion

The objective of this study was to bring up the history of the reclassifications and redefinitions of OK proposed by the WHO, and then to establish the impact of such classifications in the prevalence and epidemiology of OTs by evaluating the diagnosed cases in an Oral Pathology Service between January 1996 and December 2016.

Since the first histological definition and classification of OTs was established in 1966 by the Department of Oral Pathology of the Royal School of Dentistry, in Copenhagen, Denmark, the knowledge about these lesions has evolved continuously, and its classifications have been changing in parallel. The 1971 WHO classification contained “odontogenic tumors, odontogenic cysts and allied lesions”. In 1992, such classification was titled “odontogenic tumors”, but still included odontogenic cysts and allied lesions.^2^ In 2005, in a third edition, “odontogenic tumors” remained disclosed, although some of the “allied lesions” remained included; however, cysts were now cut out despite the extreme significance of traditional odontogenic cysts for the differential diagnosis of odontogenic tumors, such as the glandular odontogenic cyst and cystic variants of calcifying cystic odontogenic tumors.^7^ The OK was reclassified as a neoplasia and the recommendation of the new term keratocystic odontogenic tumor (KCOT) was taken.^6^ In 2017, a new classification was described and the most controverted decision was related to shifting KCOT back to the category of cyst and name it OK or OKC.

Most OTs cases belonged to patients living their second decade of life – followed by the third decade –, corroborating the studies made by Servato (2010) and Jaeger, et al. (2016).^8^^,^^9^ There was no statistically significant difference between genders and the mandible was the most affected site, with more than half of the cases belonging to this site.

The most important results of this study are related to the prevalence of lesions. Using the 2005 WHO classification (3^rd^ edition), a 287.80% increase in the prevalence of OTs was detected, considering that this result corroborates some published studies such as a 2010 study by Gaitán-Cepeda, which also aimed at establishing the frequency and prevalence of OTs before and after 2005.^10^ By utilizing files from a Mexican Histopathology Head and Neck Service, the authors demonstrated that redefining OK as a tumor led to a 92% increase in the frequency and prevalence of OTs. Another study published by Servato in 2013 showed a 50% increase in the frequency of OTs after reclassifying OK as tumor.^9^ In 2016, another similar study indicated a 464.2% increase in the prevalence of OTs when the 3^rd^ edition was used for OT reclassification.^8^^,^^9^^,^^10^ Despite the obvious differences between the obtained percentages from one study to another, every report demonstrated significant increases regarding OTs prevalence when using the 2005 classification. In addition, our study showed that when using the 1992 version (2^nd^ edition), odontoma was the most frequent OT; however, when applying the 2005 version (3^rd^ edition), KOT became the most frequent OT. These data agree with similar previously published studies.^8^^,^^9^^,^^10^ One possible explanation for both results is the inclusion or exclusion of OK in OT. Probably, this is the most important cause to such increase in 2005, and change in the most prevalent lesion.

In 2005, the most debated decision of WHO was the reclassification of OK as a neoplasia and the recommendation of the new term keratocystic odontogenic tumor (KCOT).^6^ To justify such reclassification, authors have emphasized the aggressive behavior, recurrence rate and occasional presence of a solid variant, as well as PTCH gene mutations in such lesion. Nevertheless, those mutations were found in syndromic patients and the six WHO references in non-syndromic cases included syndromic patients. Numerous subsequent studies showed PTCH mutations in approximately 85% of OKs in syndromic patients, against 30% in non-syndromic patients. However, mutations are non-clonal or limited to PTCH, since p16, P53, MCC, TSLC1, LTAS2 and FHIT mutations were also related in OKs. Although neoplasia is characterized by genetic aberrations, there are currently no unique genetic alterations to define neoplasia. The molecular/genetic modification that happens to some OKs may influence their biologic behavior but still not characterize the lesion of neoplastic rather than cystic origin. Neoplasia show growth autonomy and do not involute spontaneously. OKs, on the other hand, have been well-documented following regression after decompression; furthermore, orthokeratinized OKs and dentigerous cysts contained PTCH mutations as well. Cutaneous cysts in syndromic and non-syndromic patients are histologically identical to OKs but classified as cysts instead of tumors. In 2005 (3^rd^ edition), WHO authors chose to classify all ghost cell lesions as neoplasia. The solid neoplastic variant was suggested to be named Ghost Cell Dentinogenic Tumor, and the cystic variant Calcifying Cystic Odontogenic Tumor.^11^^,^^17^

Several changes were considered and incorporated so a contemporary consent could provide the world head and neck pathology community with an infrastructure to support the diagnoses of odontogenic cysts, odontogenic tumors and allied osseous tumors. Odontogenic cysts that were omitted from the 2005 classification were reincorporated in 2017 (4^th^ edition) and significantly updated after the 1992 classification.^18^ The subdivision of benign OTs in the 2017 classification was changed regarding its nomenclature, which was justified by the authors as a simplification. Benign OTs were thus subdivided into odontogenic epithelium tumors, odontogenic epithelium, mesenchyme and mesenchymal tumors.^6^ The most controverted decision of 2017 regarded to shifting KOT back to the category of cyst and name it OK. It is worth noting that the WHO consent does not affirm that OKs are not of neoplastic origin, although it believes that there is a lack of support to justify OKs as tumors.^11^^,^^16^^,^^18^ Other lesions that were excluded or included with 2017 classification could also influence the epidemiology of OTs, although less significantly than OK as those other entities are rare. In 2017, the authors unanimously classified the calcifying cystic odontogenic tumor as an odontogenic cyst, and a 2008 study analyzed the WHO classification about ghost cells, suggesting the need for complementary studies to unravel their biological behavior. The same authors pointed out that less than 90% of all ghost cell lesions are entirely cystic or related to odontomas, lesions that are not classified as tumors whatsoever.^18^^,^^19^ Some examples of the 2017 classification modification are the inclusion of odontogenic carcinoma, reinsertion of odontogenic carcinosarcoma, exclusion of odontoameloblastoma, the description of a new OT referred in 2014 as the primordial odontogenic tumor.^18^^,^^20^^,^^21^ Moreover, for the 2017 edition, the authors decided to group both ameloblastic fibrodentinoma and ameloblastic fibro-odontoma under the odontoma section. However, some ameloblastic fibromas do not produce hard tissues and may be regarded as true neoplasia once leading to mineralized odontogenic tissue, they probably evolve into odontomas and thus are classified such.^18^^,^^22^^,^^23^

## Conclusion

We identified a significant increase (287.80%) in the prevalence of OTs when the 2005 (3^rd^ edition) was used. Such finding corroborates with similar studies that were conducted previously and may be explained by the fact that OK was considered an OT in 2005. While this classification of odontogenic lesions might be considered as an academic exercise, the research process and updates involved are crucial for the correct diagnosis and treatment imposed.
